# The MedPhys match survey: Search criteria and advice for programs and applicants

**DOI:** 10.1002/acm2.13235

**Published:** 2021-03-30

**Authors:** Kristi R. G. Hendrickson, Titania Juang, Anna E. Rodrigues, Jay W. Burmeister

**Affiliations:** ^1^ Department of Radiation Oncology University of Washington Seattle WA USA; ^2^ Department of Radiation Oncology UC San Diego La Jolla CA USA; ^3^ Department of Radiation Oncology Duke University Medical Center Durham NC USA; ^4^ Karmanos Cancer Center Gershenson Radiation Oncology Center Wayne State University School of Medicine Detroit MI USA

**Keywords:** medical physics residency, MedPhys match, residency search process

## Abstract

**Purpose:**

The purpose of this study was to gauge the experiences of applicants and program directors (PDs) in the Medical Physics (MedPhys) Match (MPM) and to determine the most important characteristics and factors that influence decision‐making for applicants and programs when screening, interviewing, and ranking in the MPM. Opinions were also solicited from applicants and PDs on the status of medical physics residencies and the selection process, such as the availability of residency positions and satisfaction with the match process.

**Methods:**

A survey was sent to all applicants registered for the 2015–2018 MPM and to all PDs registered for the 2015–2017 MPM. Survey questions asked about the pre‐interview screening, interview, and ranking stages of the residency match process. Survey data were analyzed using graphical methods and spreadsheet tools.

**Results:**

An increasing percentage of applicants are female and/or hold a PhD as their highest degree. The over all number of interview invitations per applicant has increased, leading some applicants to decline interviews with the top reasons being cost of travel and scheduling conflicts. The top considerations for applicants in ranking programs were residency program/institution reputation, program structure/organization, and facilities/equipment available. The primary considerations identified by PDs for ranking applicants included impressions from the interview, personality fit, and clinical potential. While two‐thirds of applicants agreed or strongly agreed with the statement that a residency position was difficult to obtain, roughly one‐third of PDs agree that the current residency placement rate is a problem.

**Conclusion:**

Four years of survey data on the experiences of applicants and PDs participating in the MPM is useful to future participants navigating the residency match system. It is hoped that the data will be helpful to inform improvements and to enhance understanding of the residency match system and how it shapes our profession.

## 1 INTRODUCTION

On August 7, 2014, the American Association of Physicists in Medicine (AAPM) and Society of Directors of Academic Medical Physics Programs (SDAMPP) announced the creation of the Medical Physics Matching Program,^1^ now known as the MedPhys Match (MPM).^2^ The MPM is similar to matching processes used by medical residency programs and provides similar potential advantages. The first match cycle began in the fall of 2014 for residency positions beginning on or around July 1, 2015, and the MPM completed its sixth cycle in 2019–2020.

Quantitative statistics on the results of the first five cycles of the MPM have been released publicly by National Matching Services, Inc. (NMS), which operates the MPM,^2^ and by other limited sources.^3^, ^4^, ^5^, ^6^ Data on the experiences of both applicants and program directors participating in the MPM, however, has not been available. This information is valuable to gauge perceptions of the process from both sides and to give both applicants and program directors useful insight.

A survey of MPM participants was developed to evaluate discriminatory behavior and ethical violations within the residency interview and match process and to gather data on the interview and match experiences of applicants and program directors. The ethical and discriminatory results from the survey have been previously published.^7^ The quantitative and qualitative data on interview and match experiences from the first four years of the MPM survey are presented here. Addressing identified concerns and implementing suggestions provided within this survey can lead to better transparency and understanding of the match process and an improved experience for both applicants and programs. In addition, knowledge of the perspectives revealed by each of the survey groups will be beneficial to the other and can enhance the success of each in navigating this high‐stakes process.

## 2 METHODS

This survey study was determined to be exempt by the Institutional Review Board of the University of Washington Human Subjects Division of the Office of Research. The anonymous and voluntary survey was then emailed to all applicants and program directors registered for the MPM. In the fourth year of the study, only applicants were emailed survey invitations, and program directors were not surveyed, as it was suspected that we were accruing responses to the same questions from the same program directors each year. An applicant was defined as an individual who had registered for the MPM, and a program director was defined as an individual who had registered their program with the MPM.

The applicant surveys consisted of 57–81 questions assessing demographics, interview offers and logistics, considerations for ranking, interview and post‐interview reflection, ethics, and match experience. Additional questions were included after the inaugural match year to address the experiences of repeat applicants. The program director surveys consisted of 35–37 questions assessing general program information, considerations for selecting candidates for interview and ranking, interview and post‐interview reflection, match experience, and evaluation of the current status of residencies in medical physics. Additional questions were added in subsequent years to address changes from the previous cycle experience.

The full survey instruments from the first two years of the survey were published as a supplement to a previous publication on ethical behaviors within the match.^7^ Question types included multiple choice, select all that apply, and free response. Responses to the questions regarding opinions were collected using a 5‐point Likert scale. Research Electronic Data Capture (REDCap) was used to collect and manage the study data.^8^ Summary statistics were used to evaluate the survey responses and were determined using functions available in a spreadsheet application (Excel 2010, Microsoft, Redmond, WA).

## 3 RESULTS AND DISCUSSION

The response rates for all surveys ranged from 28 to 33% for applicants and 48‐61% for program directors, as shown in Table 1. The response rates for applicants are typical of other medical physics survey studies. The higher than normal response rate for PDs increases the validity of the data presented. According to statistics published by NMS, the percentages of registered MPM applicants who submitted a rank list were 70%, 63%, 77%, and 70%^2^ compared to our survey respondent data of 84%, 83%, 92%, and 89% in the survey years 2015 through 2018 (see Table 4). The percentages of matched applicants were 27%, 32%, 37%, and 43% (official MPM demographics and including all registrants) compared to our 48%, 67%, 64%, and 43%, in the survey years of 2015 through 2018 (see Table 2). Our survey results show a bias towards respondents who submitted ranks lists and matched, as the survey matched applicant rate is consistently higher than that of the MPM demographics. However, since the results from this survey provide important data to applicants seeking a successful match, this bias may be useful.

**Table 1:** Number of questions and the survey response rate for the applicants’ and program directors’ survey for each year.

	Applicants’ survey		Program directors’ survey	
	No. of questions	Response rate	No. of questions	Response rate
2015	57	111/402 = 28%	35	42/79 = 53%
2016	63	101/331 = 31%	36	47/77 = 61%
2017	70	91/291 = 31%	37	40/84 = 48%
2018	81	90/272 = 33%	NA	NA

**Table 2:** Demographic distribution of applicant survey responses. Absolute values indicated in parentheses. Blanks indicate that the question was not included in that survey. MPM‐reported statistics are from MPM website.^2^

	2015	2016	2017	2018
Gender				
Male	73% (73)	64% (64)	58% (53)	61% (55)
Female	32% (32)	33% (33)	38% (34)	37% (33)
Declined to respond	4% (4)	4% (4)	4% (4)	2% (2)
Ethnicity				
White‐Caucasian	64% (69)	51% (50)	62% (55)	55% (49)
Asian	17% (18)	27% (27)	16% (14)	26% (23)
Hispanic‐Latino	6% (7)	6% (6)	4% (4)	9% (8)
Black‐African American	4% (4)	3% (3)	6% (5)	2% (2)
Other	2% (1)	4% (4)	6% (5)	3% (3)
Declined to respond	7% (8)	9% (9)	7% (6)	4% (4)
Citizenship				
US citizen	75% (82)	59% (59)	59% (52)	69% (61)
Foreign citizen	10% (11)	17% (17)	20% (18)	11% (10)
US permanent resident	7% (8)	9% (9)	6% (5)	7% (6)
Canadian citizen	7% (7)	9% (9)	13% (11)	10% (9)
Other	0% (0)	5% (5)	0% (0)	1% (1)
Declined to respond	1% (1)	1% (1)	2% (2)	1% (1)
Education				
PhD	–	54% (60)	62% (56)	67% (60)
MS	–	41% (29)	36% (33)	32% (29)
Declined to respond	–	–	2% (2)	1% (1)
PhD medical physics	–	–	35% (32)	39% (35)
MS medical physics	–	–	39% (35)	39% (35)
Certificate medical physics	–	–	19% (17)	16% (14)
No medical physics degree	–	–	7% (6)	4% (4)
Declined to respond	–	–	1% (1)	2% (2)
CAMPEP accredited degree	Yes		90% (82)	89% (75)
	No		2% (2)	11% (9)
	No response		8% (7)	7% (6)
Specialty				
Therapy	–	71% (72)	65% (59)	74% (67)
Imaging	–	6% (6)	8% (7)	10% (9)
Therapy and imaging	–	18% (18)	25% (23)	14% (13)
Declined to respond	–	5% (5)	2% (2)	1% (1)
Match results				
Matched	48% (52)	67% (68)	64% (58)	62% (56)
Unmatched	51% (56)	29% (29)	31% (28)	37% (33)
Declined to respond	1% (1)	4% (4)	5% (5)	1% (1)
MPM reported statistics				
Matched	27%	32%	37%	43%
Unmatched	73%	68%	60%	57%

### 3.1 Applicant survey results

The demographic distribution of survey respondents is shown in Table 2, including the education level reported by respondents. More detailed questions regarding highest level of education vs highest level of medical physics education and whether programs were CAMPEP accredited were asked in 2017 and 2018 surveys only. To the extent that survey respondents are representative of the residency applicant pool, these results show an increasing percentage of female applicants and variable percentages of ethnically diverse applicants. The percentage of applicants with highest degree as PhD is increasing from 54% in 2016 to 62% and 67% in 2017 and 2018, respectively. Correspondingly, the percentage of applicants with MS as highest degree is decreasing.

### 3.2 Number of applications

**Fig. 1:**
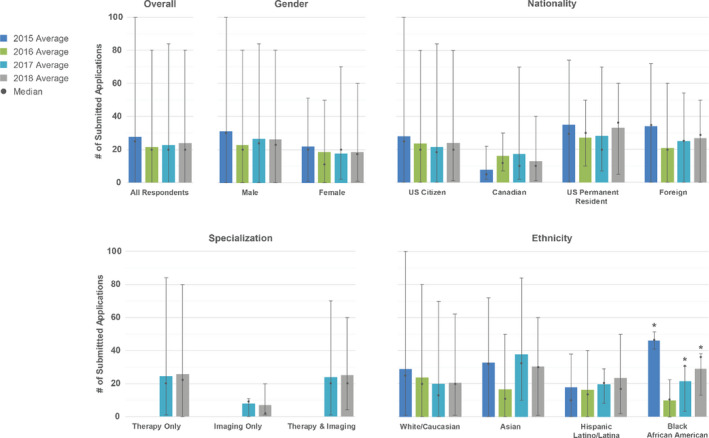
Distribution of number of submitted applications per applicant respondent for each year. The over all distribution is shown in the top left plot. Subsequent plots break down the distribution as a function of demographics (gender, nationality, specialization, and ethnicity). Bars indicate the average number of applications submitted per respondent, whereas the dots indicate the median. The error bars indicate the range (minimum to maximum) of submitted application numbers. Asterisks (*) indicate that the total number of respondents included in the data shown is ≤3.

Applicants were asked how many applications they submitted. In the first year of the MPM, the match was supported financially by AAPM and SDAMPP, and therefore there was no registration fee for applicants. Starting with the 2017 MPM, AAPM and SDAMPP reduced their subsidization, with the balance of funding supported by fees from applicants ($55) and programs ($175). Starting with the 2019 MPM, full funding was obtained from applicants ($100) and programs ($375). Additionally, many programs utilized MP‐RAP for collecting applications, which was free in the inaugural year of the match only. Results related to numbers of applications submitted are shown in Fig. 1. Over all, the average number of applications submitted per applicant has decreased from 2015 and leveled off to about 20 in subsequent years. The general trend by gender is that females submit fewer applications than their male counterparts, and U.S. citizens submit fewer applications on average than US permanent residents and foreign applicants, although there have been individuals who submit ≥ 80 applications per year, close to the total number of programs offering positions in the match each year. Therapy applicants submit on average twice as many applications as imaging applicants, which may be largely due to fewer imaging residency positions offered each year compared to therapy positions.

### 3.3 Interviews, rank lists, and preferences

**Fig. 2:**
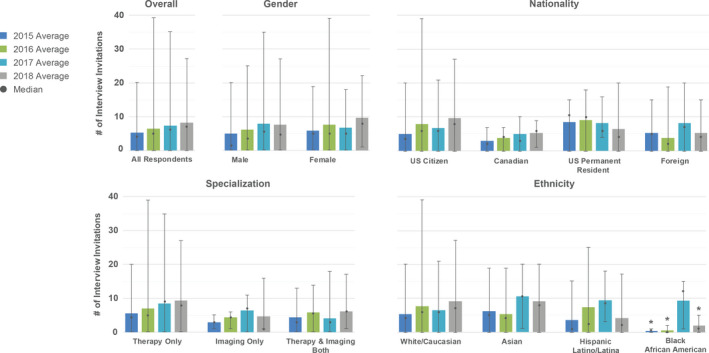
Distribution of number of interview invitations received per applicant respondent for each year. The over all distribution is shown in the top left plot. Subsequent plots break down the distribution as a function of demographics (gender, nationality, specialization, and ethnicity). Bars indicate the average number of interview invitations received per respondent, whereas the dots indicate the median. The error bars indicate the range (minimum to maximum) of interview invitations. Asterisks (*) indicate that the total number of respondents included in the data shown is ≤3.

**Fig. 3:**
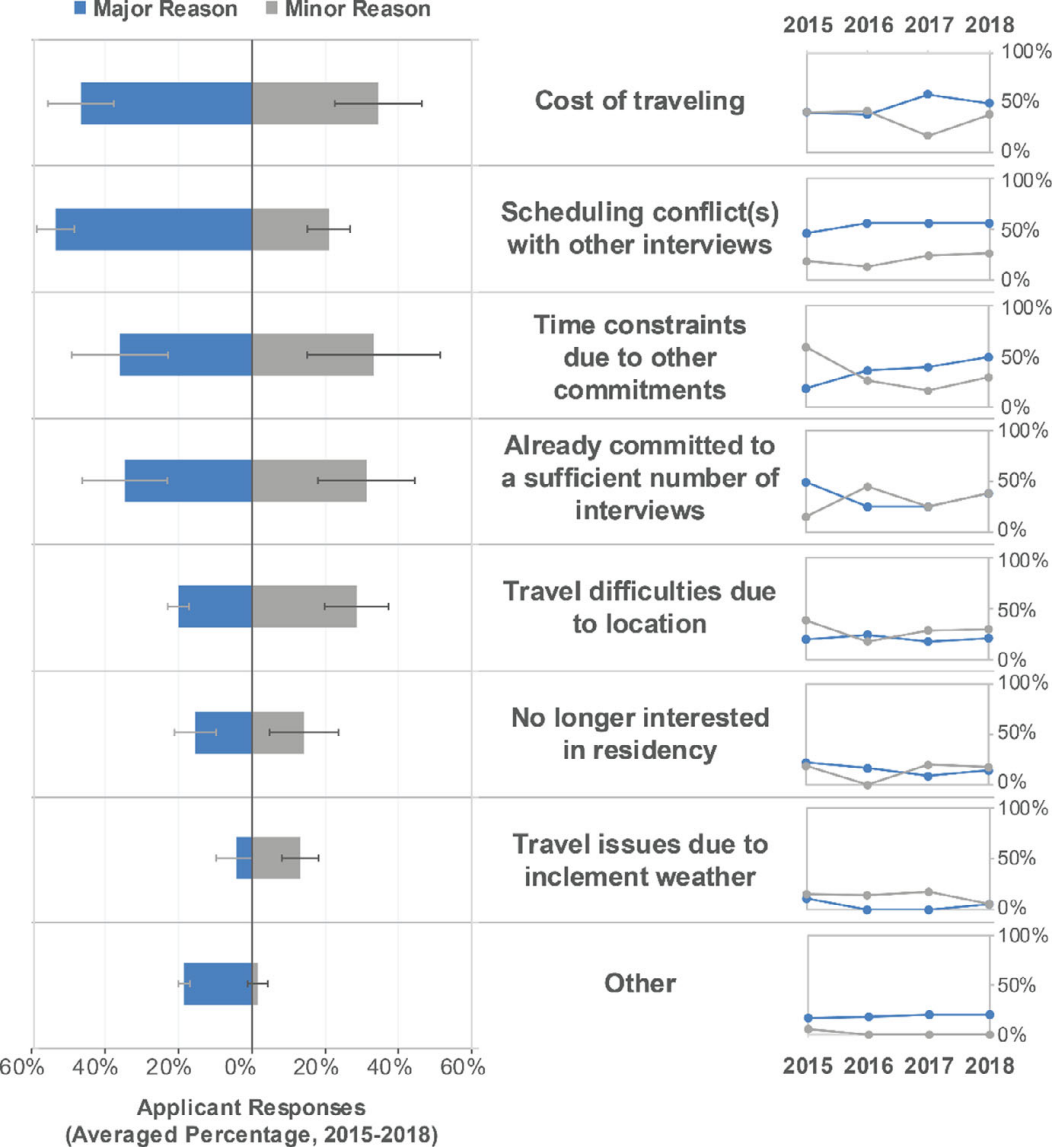
Applicants' reasons for declining interview invitations. Middle column: Reasons ranked highest to lowest on average over all years where the choices included “major reason,” “minor reason,” and “not a reason.” Left column: A double‐sided bar graph displays average response over all years listing major reason or minor reason. Error bars on the averaged percentages indicate standard deviation. Right column: Scatter plot for major and minor reasons across each year of the survey to show the changing importance of each reason category over time.

Applicants were asked how many interview invitations they received and whether they declined any interviews that were offered. If a respondent indicated that they declined any interviews, they were further asked to specify the reason(s) for declining. Several possible reasons were offered, and respondents chose whether each possibility was a Major Reason, Minor Reason, or Not a Reason. A text box was available to indicate additional reasons for declining an interview. Results related to interviews are shown in Figs. 2 and 3. The over all average and median number of interview invitations per applicant increased from 2015 to 2018. Looking at the data filtered by nationality, the average number of interview invitations per resident decreased for US permanent residents while tending to increase for US citizen and Canadian applicants.

Applicants were asked how many interviews they attended or participated in and whether the interview was on‐site or conducted remotely, such as by telephone or videoconference. Table 3 shows the total number of interviews attended and the percentage of interviews that were in person vs conducted remotely. For example, in 2018, 69% of all interviews were on‐site, accounting for 548 interviews, and 30% (242 interviews) were conducted remotely. Of those 242 remote interviews, 81% were reported to be screening interviews, and 19% were final interviews. Similar numbers are reported for other available years. While applicants received an average and median number of interview invitations < 10 per year, some individual applicants received 20‐40 interview invitations, necessitating that some applicants decline some interview invitations. As shown in Fig. 3, the top reasons for applicants to decline on‐site interview invitations were cost of travel and scheduling conflicts with other interviews. “Time constraints due to other commitments” has risen from a minor reason in 2015 to an increasing major reason in subsequent years of the survey. Other commitments can include scheduling thesis defense dates and other required dates related to degree completion.

**Table 3:** Total number and over all percentage of interviews conducted in person (on‐site) and remotely as reported by applicant respondents for each year. For 2017 and 2018, remote interviews are further classified as initial screening interviews or final interviews.

	In person interviews	Remote interviews		
		Over all	Screening	Final
2015	73%/389	27%/142	–	–
2016	72%/461	28%/180	–	–
2017	70%/486	30%/204	80%	20%
2018	69%/548	30%/242	81%	19%

**Fig. 4:**
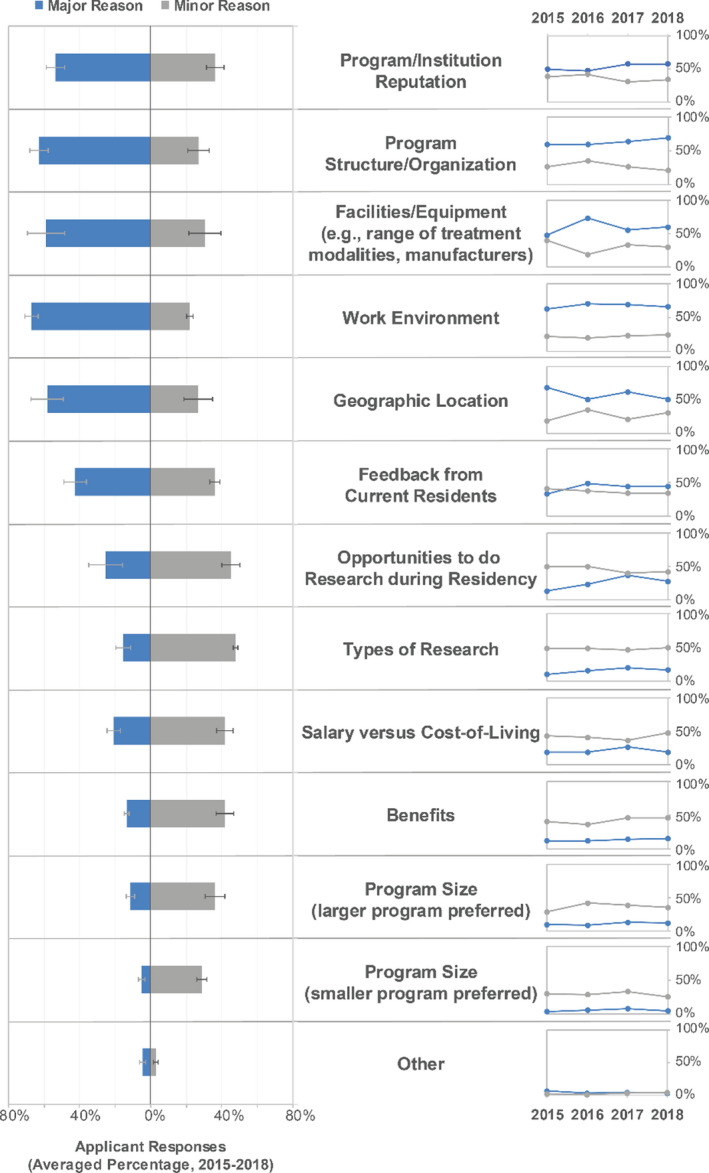
Applicants' considerations for ranking residency programs. Middle column: Reasons ranked highest to lowest on average over all years where the choices included “major reason,” “minor reason,” and “not a reason.” Left column: A double‐sided bar graph displays average response over all years listing major reason or minor reason. Error bars on the averaged percentages indicate standard deviation. Right column: Scatter plot for major and minor reasons across each year of the survey to show the changing importance of each reason category over time.

The percentage of applicant respondents who submitted a rank list in each year of the MPM is shown in Table 4 and averages to 87% over the four survey years. These values are higher than the over all percentage of applicants to the MPM who submitted a rank list (average 70%), as reported on the MPM website.^2^ Respondents who indicated that they did submit a rank list were asked in a follow‐up question to rate the importance of a variety of possible criteria for preferring one residency program over another. These data are shown in Fig. 4. The top attributes that respondents considered important when making ranking decisions about programs were residency program/institution reputation, residency program structure/organization, facilities and equipment available at the institution, the work environment, and the geographic location. The least important reasons were program size in terms of number of residents and benefits packages.

**Table 4:** Percentage of applicant respondents who submitted a rank list for each year. Over all MPM statistics were deduced from the MPM website as the percentage of applicants who submitted a rank list (i.e. “Applicants Participating in the Match”) normalized to the number of applicants who registered for the match.^2^ Survey statistics are reported both as over all percentages as well as broken down by gender and specialization.

	MPM statistics	Survey statistics					
	Over all	Over all	Male	Female	Therapy only	Imaging only	Both
2015	70%	84%	81%	94%	–	–	–
2016	63%	83%	81%	88%	87%	83%	78%
2017	77%	92%	92%	91%	93%	100%	90%
2018	70%	89%	87%	91%	88%	89%	92%

### 3.4 Match results and reapplication

**Fig. 5:**
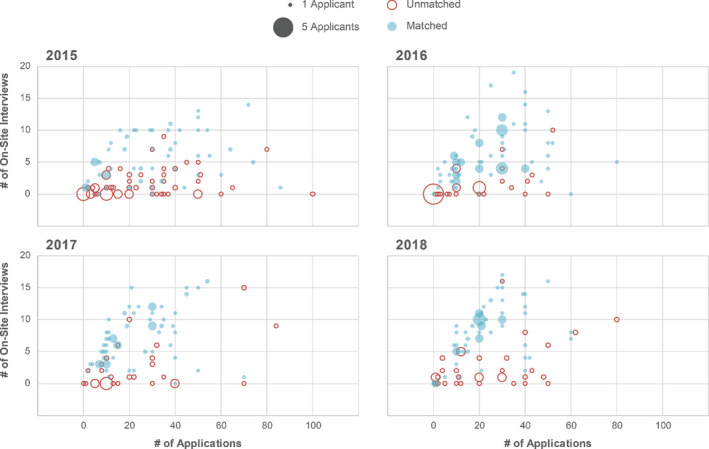
Number of applications submitted as a function of number of on‐site interview invitations for each year. Open and filled circles indicate whether the respondent was unmatched or matched in the given year, respectively. The relative size of the circle indicates the number of applicants.

Table 5 shows matching statistics for all respondents and a comparison to matching rates published by MPM. Females were more likely to match than males, while imaging‐only applicants were more likely to match than therapy only or than applicants that applied to both therapy and imaging residencies. The number of respondents in some categories is sometimes small and therefore challenging to draw statistically significant conclusions. A scatter plot of the number of applications submitted versus the number of on‐site interviews is presented in Fig. 5, including whether or not that resulted in a successful match.

**Table 5:** Percentage and absolute number of applicant respondents who matched for each year. MPM statistics were deduced from the MPM website^2^ as the percentage of applicants who matched normalized to the number of applicants who registered for the match. Survey statistics are reported both as overall percentages as well as broken down by gender and specialization.

	MPM statistics	Survey statistics					Both
	Overall	Overall	Male	Female	Therapy only	Imaging only	
2015							
Matched	27%	48%/52	45%/33	58%/18	–	–	–
Unmatched	43%	52%/56	55%/40	42%/13	–	–	–
2016							
Matched	32%	70%/68	64%/40	87%/27	69%/49	83%/5	67%/12
Unmatched	31%	30%/29	36%/23	13%/4	31%/22	17%/1	33%/6
2017							
Matched	37%	67%/58	62%/31	74%/25	74%/42	100%/6	46%/10
Unmatched	40%	33%/28	38%/19	26%/9	26%/15	0/0	54%/12
2018							
Matched	43%	63%/56	51%/28	85%/28	66%/44	67%/6	46%/6
Unmatched	32%	37%/33	49%/27	15%/5	34%/23	33%/3	54%/7

A key question for applicants is how many interviews are needed to be successful in the match? Figure 5 may serve as a guide to help answer this question. More on‐site interviews (we were unable to include the small percentage of remote final interviews) generally does result in a successful match. However, it is still possible to attend > 10 interviews and not match with a program; and it is also possible to attend < 5 interviews and successfully match. Generally, five interviews appear to be a reasonable line above which matching success is more likely. Performance during the interview is likely a key to matching success more than whether the paper application was enough to get several interview invitations. It is unclear in the survey responses what is indicated by respondents who successfully matched with zero interviews; the entry may be an inadvertent mistake on the part of the survey respondent.

**Fig. 6:**
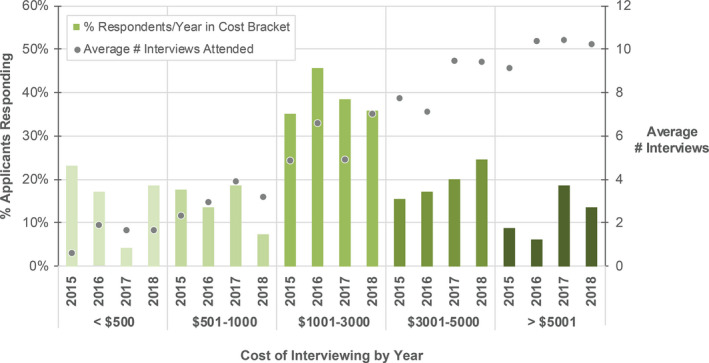
Total cost of interviewing categorized by range <$500, $501–1000, $1001–3000, $3001–5000, and >$5001 (left to right) for each year. The percentage of respondents (left y‐axis) for each year is shown as a bar. A scatter plot is superimposed indicating the average number of interviews (right y‐axis) attended per year and cost category.

Applicants were asked the total cost of attending interviews. The results are shown in Fig. 6, including a breakdown over each year of the survey. As expected, applicants who attended more interviews spent more on interviewing costs. Applicants will note that there is no correlation between the total cost spent on interviews and success in the match. It is hoped that programs will note the substantial costs that applicants have been incurring when designing their on‐site interview plans.

It is unknown from these survey data whether there is a matching advantage for an applicant to participate in a remote final interview versus in person. However, this question is now more relevant given that residency programs pivoted to entirely remote interviewing during the 2020–2021 match cycle due to the COVID‐19 pandemic.

Unmatched survey respondents were asked if they intended to apply to the match in the future, as shown in Table 6. The percentages of applicants planning to reapply (“Yes”) ranged from 47% to 68% in the four years of the survey. Alternatives to the MPM included individuals who already accepted a position outside the match, pursuing a non‐medical physics career, and seeking a medical physics position where a residency is not required. Respondents who indicated that they intend to apply to the MPM again reported that they are preparing for reapplication with a medical physics research position such as a post doc or additional education such as a PhD. Other reported strategies to prepare for reapplication included working within a radiation oncology department to gain more skills, observing medical physics work, and authoring more publications.

**Table 6:** Percentage and absolute number of unmatched applicant respondents who reported whether they would apply again in a subsequent year.

	Yes	No
2015	47%/26	53%/29
2016	68%/19	32%/9
2017	57%/16	43%/12
2018	54%/18	46%/15

Four individuals reported that they withdrew from the match in 2018 due to acceptance of a residency position outside the match, inability to complete education in time for residency start date, or no interview offers received. The corresponding result from other survey years was six in 2017, seven in 2016, and ten in 2015. Applicants who withdraw from the match after interviewing and prior to the match deadline are of interest to PDs, who express concerns about the limitations of resources and number of on‐site interview slots that ultimately are used by applicants who withdraw. PDs speculate or try to estimate the percentage of withdrawals they will experience and might overestimate the number of applicants needed to interview in order to fill their open slots in the match. The reasons for withdrawing, such as not completing the requisite degree on time, suggest that graduate advisors and students should more carefully consider match cycle dates in relation to realistic thesis completion schedules when a student wishes to participate in the match. The practice of offering positions outside the match during the timeframe leading up to the match deadline should be avoided to minimize the number of applicants who withdraw for this reason.

**Fig. 7:**
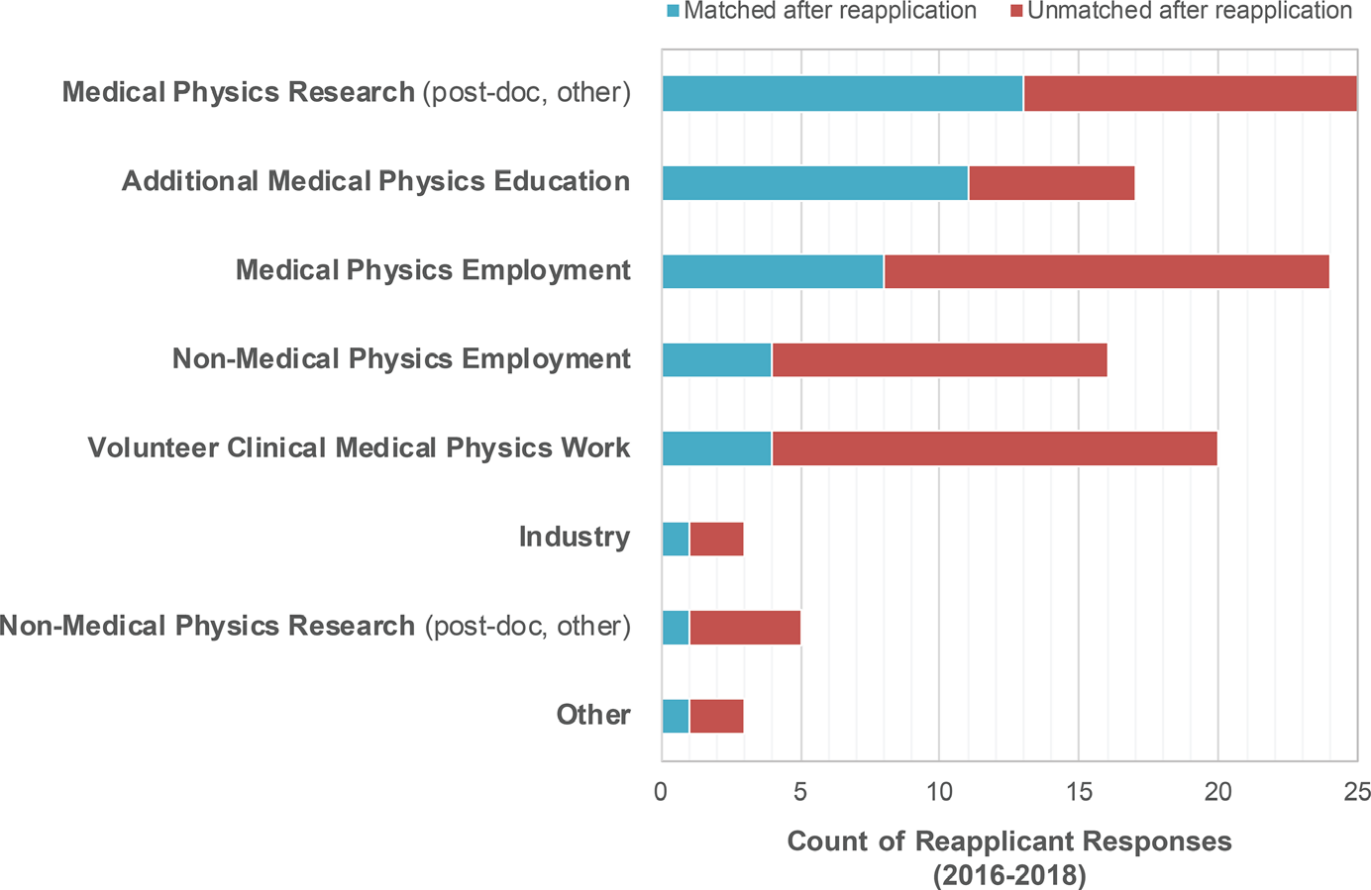
Reapplicant activities between match cycles. Data shown are total reapplicant responses for the survey years 2016–2018 and indicate matched and unmatched reapplicant respondents. Activities are ranked in descending order based on the number of respondents who matched after reapplication.

In the 2018 applicant survey, 19% (17 individuals) reported that they had applied for a medical physics residency position in a previous match cycle. Of those 19% reapplicants, 82% reported that they received interview invitations in the current year. Thirteen individuals had previously applied in 2017, four in 2016, and five in 2015, where some individuals reported that they reapplied in multiple years. Thirty‐five percent of reapplicants (six individuals; caution: small data results) were successfully matched in 2018. Reapplicants were asked what experiences or activities they pursued between match cycles. Their responses are shown in Fig. 7, and the top responses are shown and color‐coded as to whether the activity resulted in a subsequent successful match. One‐third of respondents who were later successful in matching engaged in medical physics employment; another third pursued additional medical physics education. Medical physics research was equally likely/unlikely to result in a successful match, whereas additional medical physics education was more likely to result in a successful match. Respondents who were not successful in a reapplication cycle were most often engaged in multiple activities, such as medical physics employment, non‐medical physics employment, medical physics research, and volunteer clinical work.

### 3.5 Current status of residencies in medical physics

**Fig. 8:**
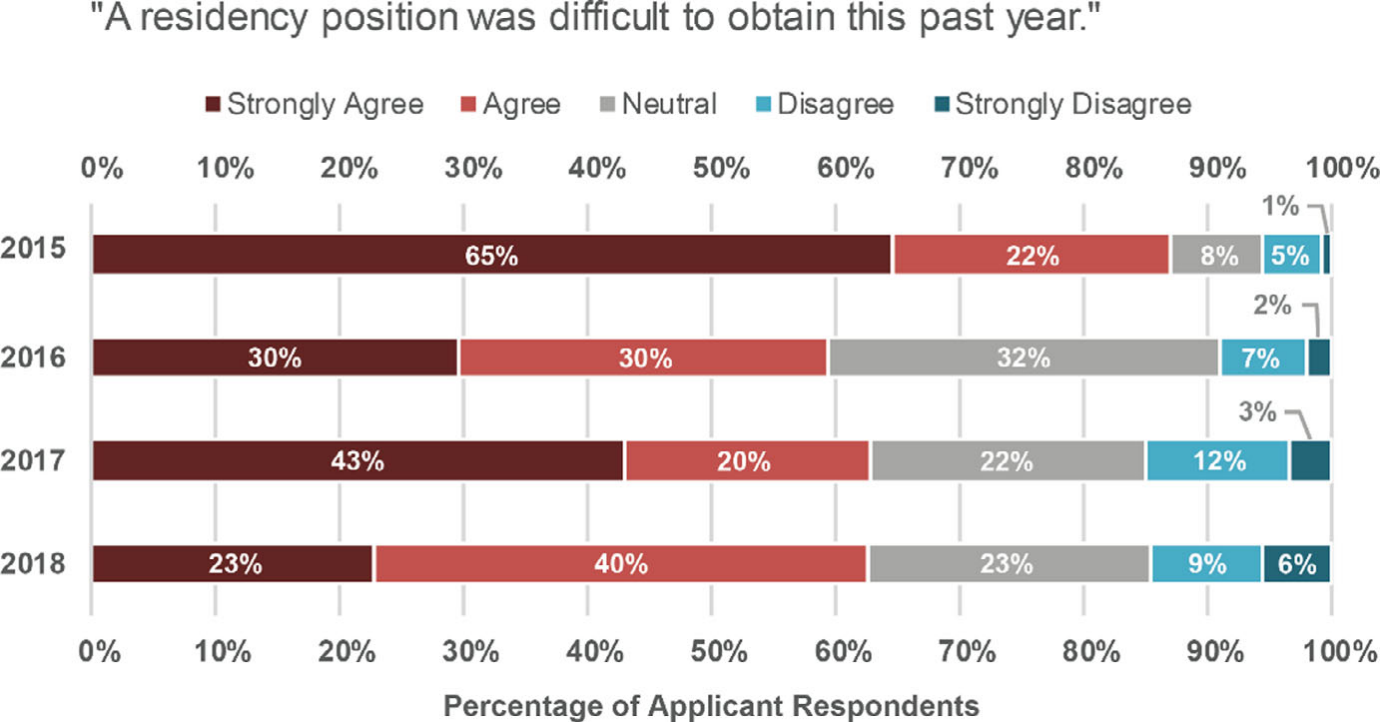
Applicant responses to the statement “A residency position was difficult to obtain this past year” for each year.

Applicants were asked whether they agreed that a residency position was difficult to obtain in their particular year, and the results are shown in Fig. 8. In 2015, 87% of applicant respondents indicated that they strongly agreed or agreed with the statement that a residency position was difficult to obtain that year. In subsequent years, the responses were 59%, 63%, and 62% in 2016, 2017, and 2018, respectively. The number of applicants who registered for the match compared to the number of positions offered in the match were as follows: 402 applicants/112 positions (2015); 331 applicants/111 positions (2016), 291 applicants/114 positions (2017), and 272 applicants/129 positions (2018). It is believed that because registration for the match was free the first year, this invited an initial influx of applicants who were not competitive for residency positions. While there are still many more applicants than residency positions available, the number of applicants to the match has decreased and stabilized in subsequent years.

**Fig. 9:**
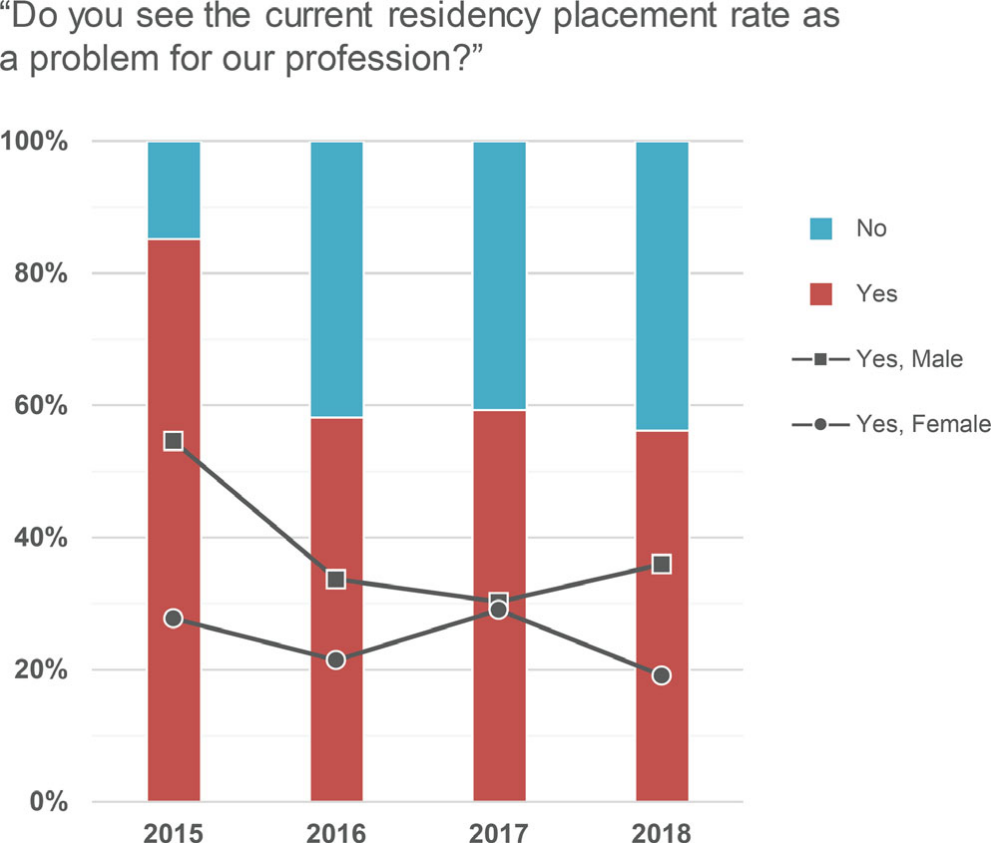
Applicant responses to the question “Do you see the current residency placement rate as a problem for our profession?” for each year.

Applicants were further asked if they see the current residency placement rate as a problem for our profession. These responses are shown in Fig. 9, where there are differences in opinions expressed by male and female respondents. Survey respondents in the inaugural year of the match, particularly male respondents at 57%, suggested that the current residency placement rate is a problem.

**Fig. 10:**
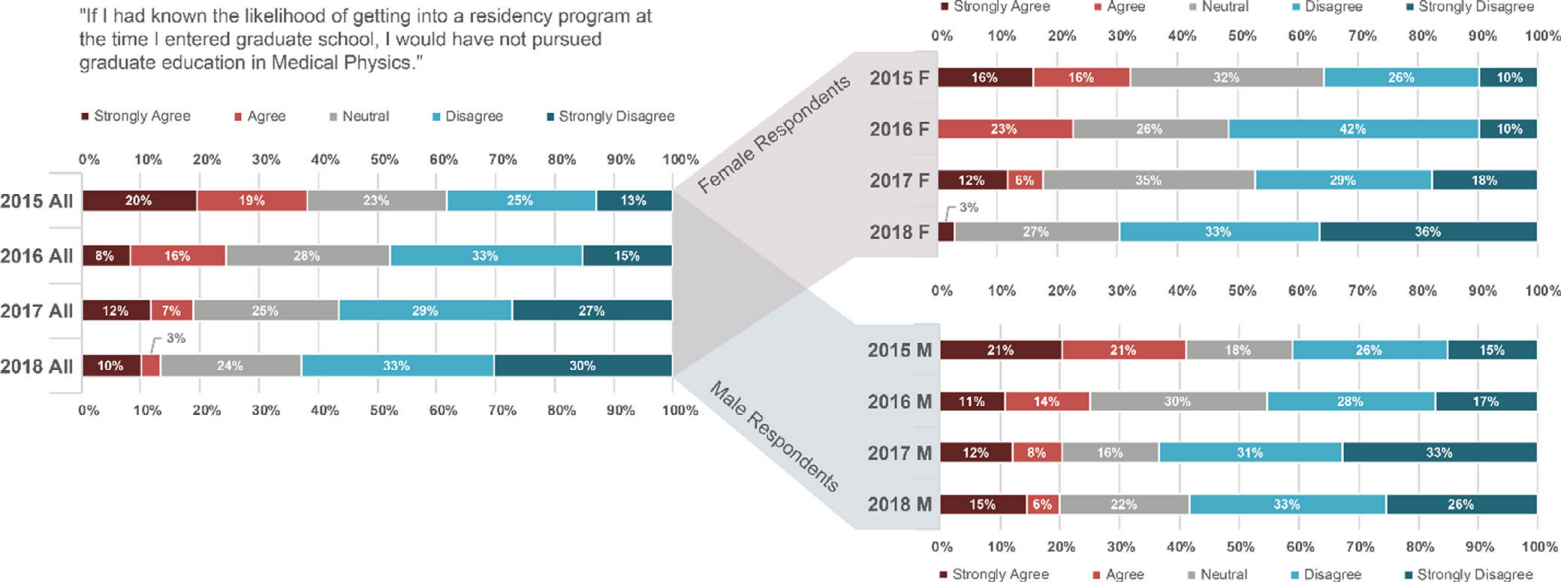
Applicant responses to the statement “If I had known the likelihood of getting into a residency program at the time I entered graduate school, then I would have not pursued graduate education in Medical Physics.” for each year. The two right bar plots further break down the responses by gender. Respondents who did not specify gender are included in the over all responses and omitted from the gender breakdowns.

Applicants were asked how strongly they agree with the statement “If I had known the likelihood of getting into a residency program at the time I entered graduate school, then I would have not pursued graduate education in Medical Physics.” Over all responses and filtered by male and female are shown in Fig. 10. Agreement with this statement was an alarming 39% in the initial 2015 survey (Fig. 10). While it has steadily decreased to 13% in 2018, this is clearly still an important consideration for assuring a continued supply of high‐quality candidates into our profession in the future.

**Fig. 11:**
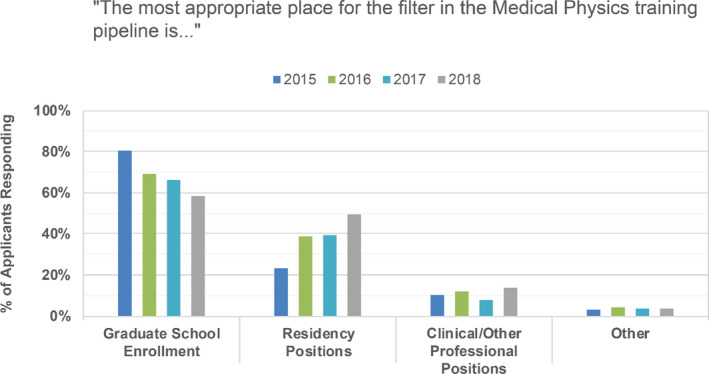
Top applicant choices in response to the statement “The most appropriate place for the filter in the Medical Physics pipeline is....”.

Applicants were asked where they believed the most appropriate place for the filter in the medical physics pipeline is. Responses are shown in Fig. 11. Note that respondents could choose more than one option. In all years of the survey, applicant respondents were more likely to choose graduate school enrollment as the appropriate filter location over residency positions, indicating that fewer graduate students should be accepted given the current number of residency slots.

### 3.6 Program director survey results

A survey of residency program experiences was sent to all program directors (PDs) who participated in the MPM in 2015, 2016 and 2017. Table 1 shows the participation rate of PDs to the survey invitation. In 2017, 78% of responding PDs were from therapy residency programs, with 22% from imaging residency programs. This question was not asked in previous survey years. Seventy‐eight percent of PDs in 2017 indicated that they have participated in the MPM all years since its inception, 20% had participated the previous year (2016), and 2% were participating for the first time in 2017. In the 2016 survey, 94% of PDs indicated that they participated in the MPM the previous year (2015).

**Fig. 12:**
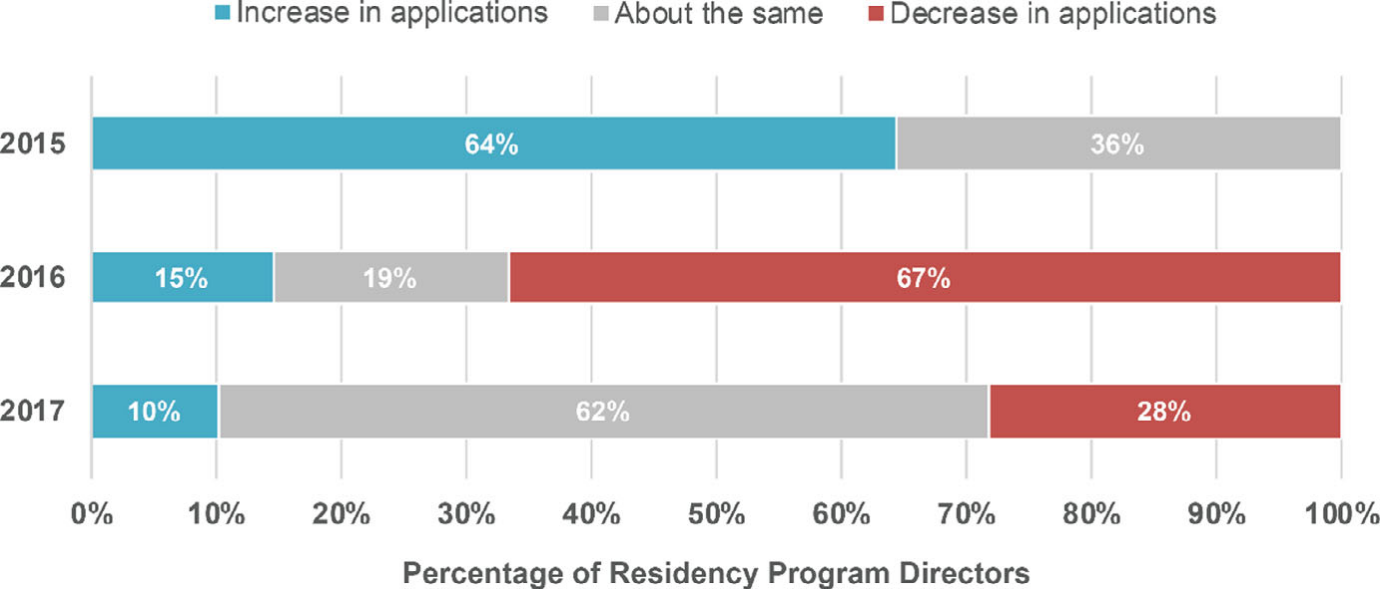
Changes in application numbers during the first three years of the MPM as reported by program directors.

PDs were asked if they experienced changes in application numbers during their participation in the MPM. Results for all three years of the PD survey are shown in Fig. 12, where initially most programs experienced an increase in applications for the first year of the match, followed by a similar decrease in the second year of the match, and finally a relative stabilization of application numbers in the third year. PDs were also asked if their program considered or interviewed MS only, PhD only, or both types of candidates, as shown in Table 7. Sixty‐nine percent considered both types of candidates, and the remaining 31% considered PhDs only. In 2017 only, PDs were asked if they offer residency positions outside of the match in addition to their participation in the match. Twelve percent of respondents indicated that they did offer at least one position outside the match. The reasons given included a starting date that did not fall between June 1 and December 31 and uncertain funding for the position.

**Table 7:** Program directors’ response to the question “Is your program open to applicants with MS only, PhD only, or both types of degrees?” for the first three years of the survey.

	2015	2016	2017
Consider MS only	–	–	0%
Consider PhD only	–	–	31%
Consider both	–	–	69%
Interview MS only	0%	2%	0%
Interview PhD only	43%	42%	28%
Interview both	57%	57%	72%

### 3.7 Interviews, rank lists, and preferences

**Fig. 13:**
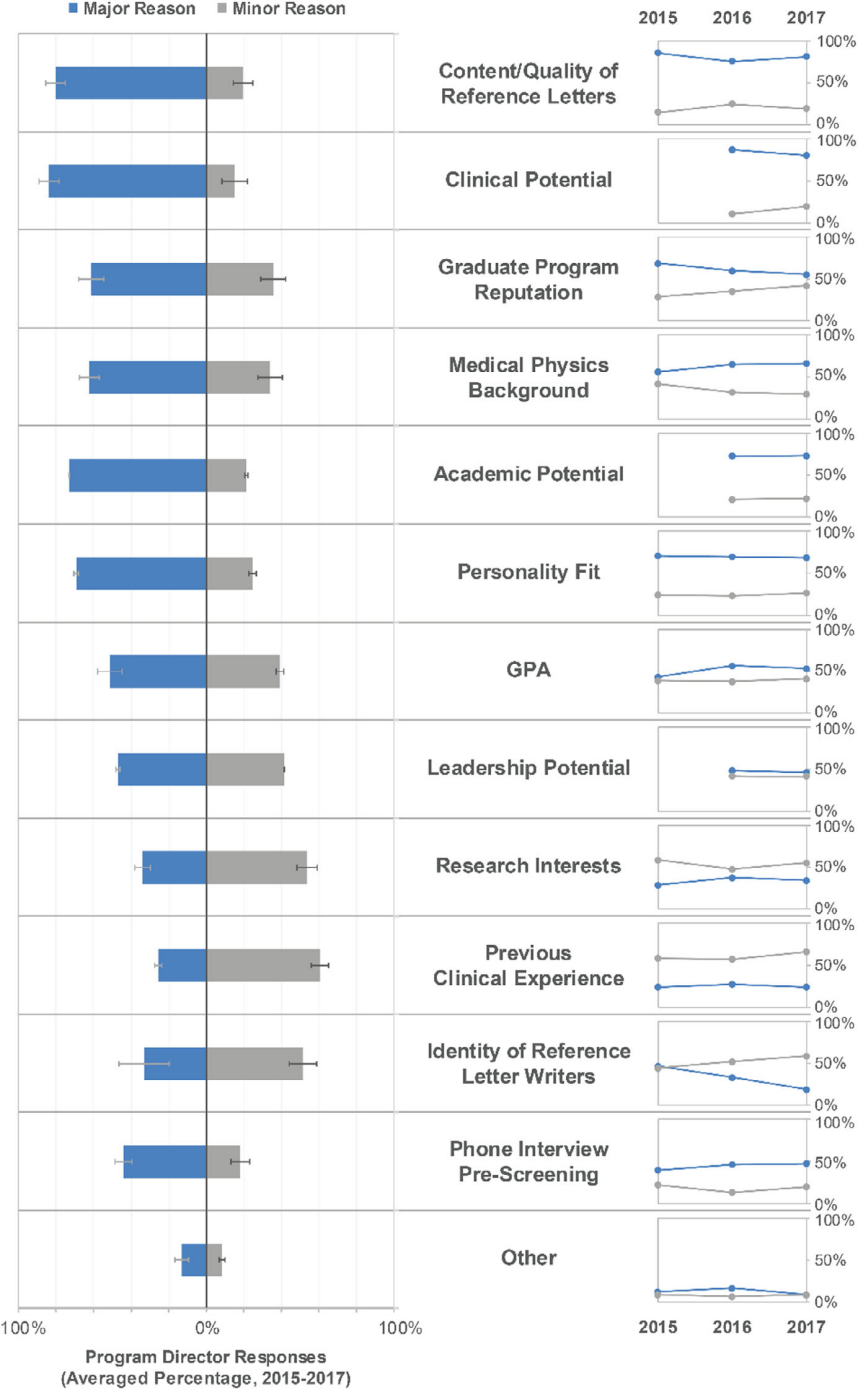
Program directors’ considerations that most influenced their choice of candidates to invite for interview. Middle column: Reasons ranked highest to lowest on average over all years where the choices included “major reason,” “minor reason,” and “not a reason.” Left column: A double‐sided bar graph displays average response over all years listing major reason or minor reason. Error bars on the averaged percentages indicate standard deviation. Right column: Scatter plot for major and minor reasons across each year of the survey to show the changing importance of each reason category over time.

PDs were asked if they considered and if they interviewed MS only, PhD only, or both degrees in their search process (see Table 7). Zero to one PD respondent to the survey per year indicated that they only consider or interview MS degree candidates. Forty‐three percent, 42%, and 28% of PDs indicated that they interview only PhD candidates. The majority of programs indicated that after considering applications, reference letters, and screening interviews, they interview both MS and PhD applicants. As shown in Fig. 13, primary considerations for interview invitation identified by PDs (with average percentage of respondents indicating this as a major factor in parentheses) included clinical potential (84%), content/quality of reference letters (80%), academic potential (73%), personality fit (69%), medical physics background (62%), and graduate program reputation (61%).

**Fig. 14:**
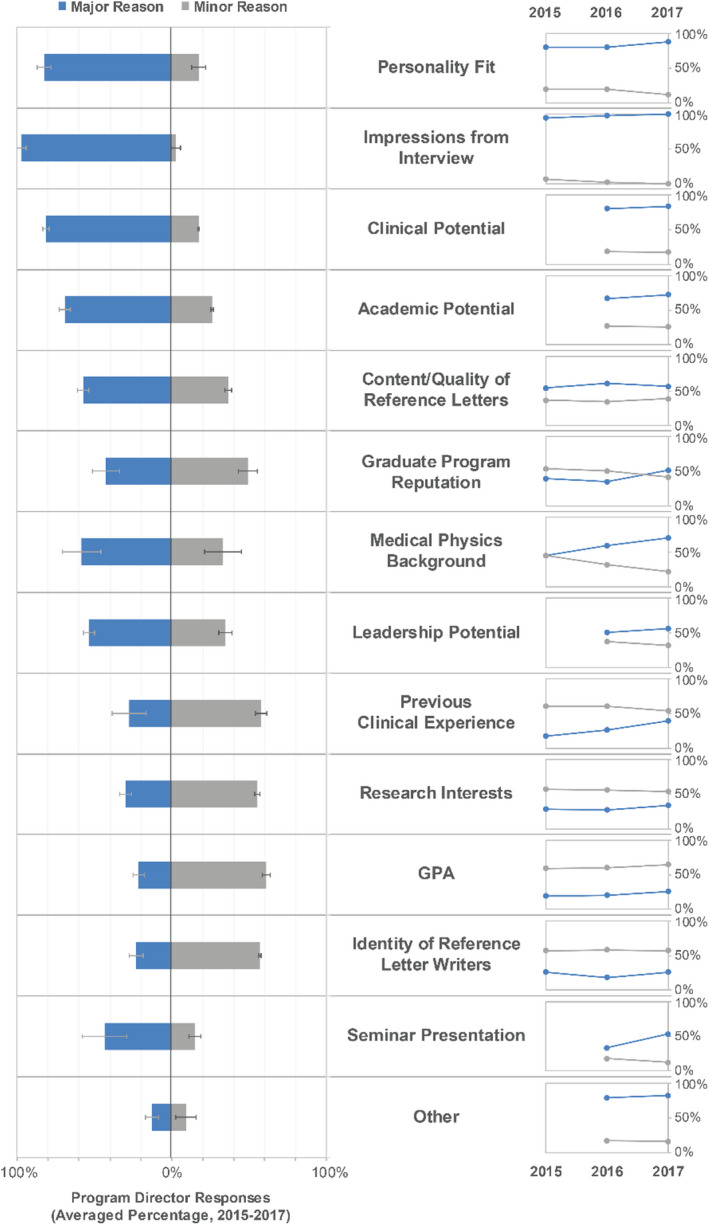
Program director considerations that most influenced final candidate rankings submitted to the match. Middle column: Reasons ranked highest to lowest on average over all years where the choices included “major reason,” “minor reason,” and “not a reason.” Left column: A double‐sided bar graph displays average response over all years listing major reason or minor reason. Error bars on the averaged percentages indicate standard deviation. Right column: Scatter plot for major and minor reasons across each year of the survey to show the changing importance of each reason category over time.

Post‐interview, primary considerations for final ranking identified by PDs (with average percentage of respondents indicating this as a major factor in parentheses) included impressions from interview (97%), personality fit (83%), clinical potential (82%), academic potential (69%), medical physics background (59%), and content/quality of reference letters (57%), as shown in Fig. 14. Other responses offered in a free text box included motivation and drive, work ethic and professionalism, communication skills, underrepresented minority, and writing abilities and interest in medical physics as expressed in a required personal essay. In summary, while graduate program reputation and reference letters are primary drivers in getting a candidate an interview, perceptions from the interview itself are the primary drivers of match ranking.

PDs were asked in 2017 about candidates who have not completed their graduate degree at the time of the match but were presumed to complete before the residency start date. Ninety percent of respondents indicated that they did rank applicants who had not yet completed their graduate degree, however, 78% said they seek or require assurances from the candidate that they will complete the degree by the start date, and 42% said they seek an attestation from the graduate program director or thesis advisor. The majority (60%) of PDs stated that the possibility that an applicant may not complete their degree prior to the program start date is a significant consideration in ranking the applicant.

PDs were asked what their recourse would be if they matched with an applicant who does not complete their degree by the program start date. Thirty‐seven percent said that they would modify the start date to allow completion of the degree, 32% said they would allow the student to begin the residency while completing the degree, and another 32% selected “Other.”

**Fig. 15:**
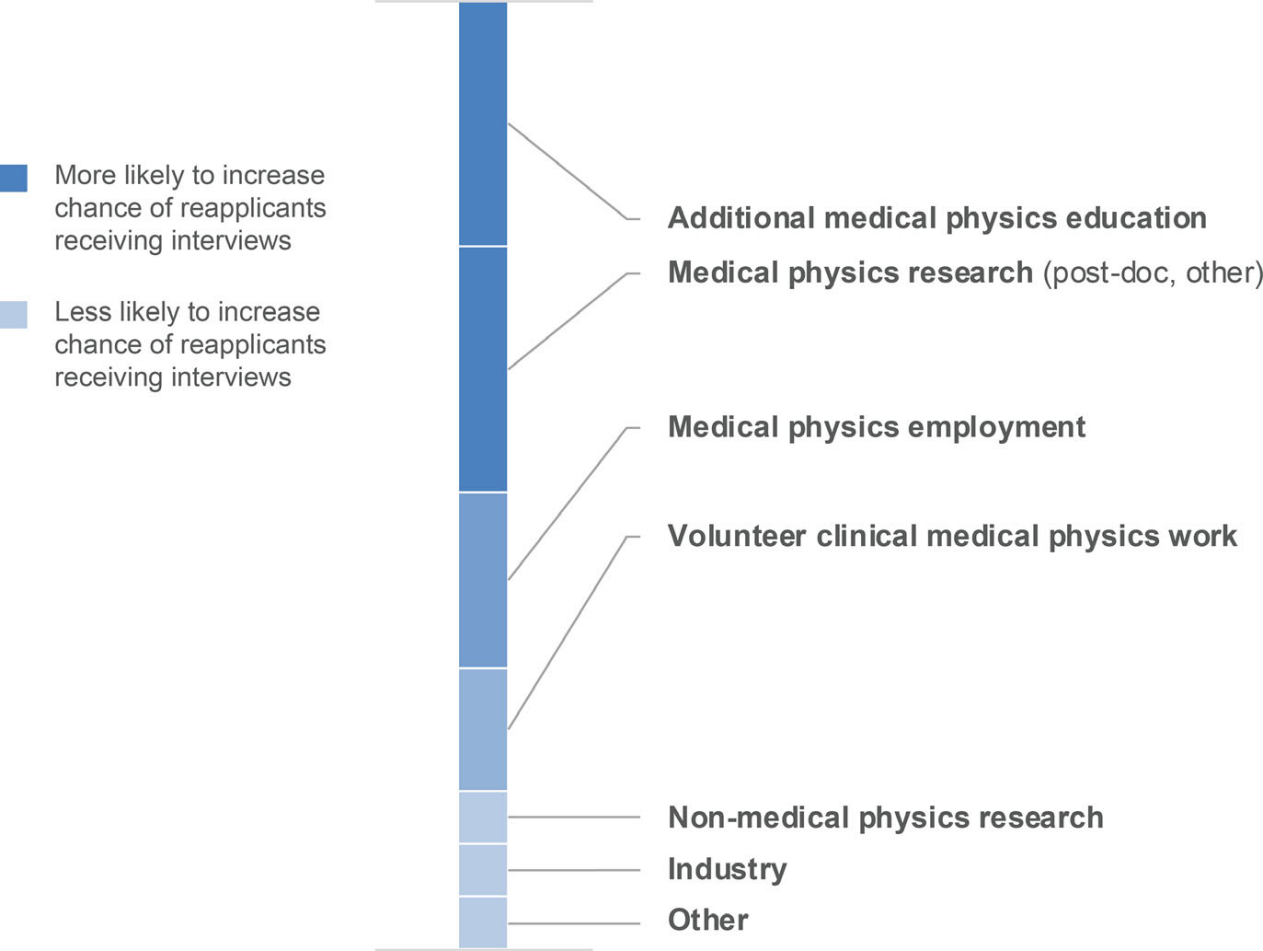
Program directors’ responses on reapplicant activities between match cycles that were most likely to increase the reapplicants’ chance of getting an invitation to interview. The size of each segment of the stacked column indicates the relative number of times a response was chosen in the 2015–2017 surveys.

In 2017, PDs were asked if they considered or interviewed reapplicants during their selection process; 54% responded yes, 15% responded no, and 32% indicated that they did not know if the candidates were reapplicants. PDs who did consider or interview reapplicants were further asked what activities in the year(s) between match cycles would most increase the applicant’s chance of getting an invitation to interview. Figure 15 shows the responses, where PDs were asked to choose all that apply. For programs that did consider reapplicants, additional medical physics education, research, or clinical experience (paid or volunteer) were the primary suggestions from PDs for candidates who did not match but wished to enter the match again the following year. The top two suggestions from PDs (additional medical physics education and medical physics research) correlate with the activities most likely to result in a subsequent residency match as reported by reapplicants in Fig. 7.

Visa requirements can affect applicant selection as some institutions are unable to accommodate certain types of visas. For example, US employment law only requires that a person needs to have valid work authorization.^9^ PDs were asked if they accept visa applicants; 29% of respondents indicated that they accept all visa types, 46% reported that their response depends on the visa type, and 24% indicated that they do not accept visa applicants.

**Fig. 16:**
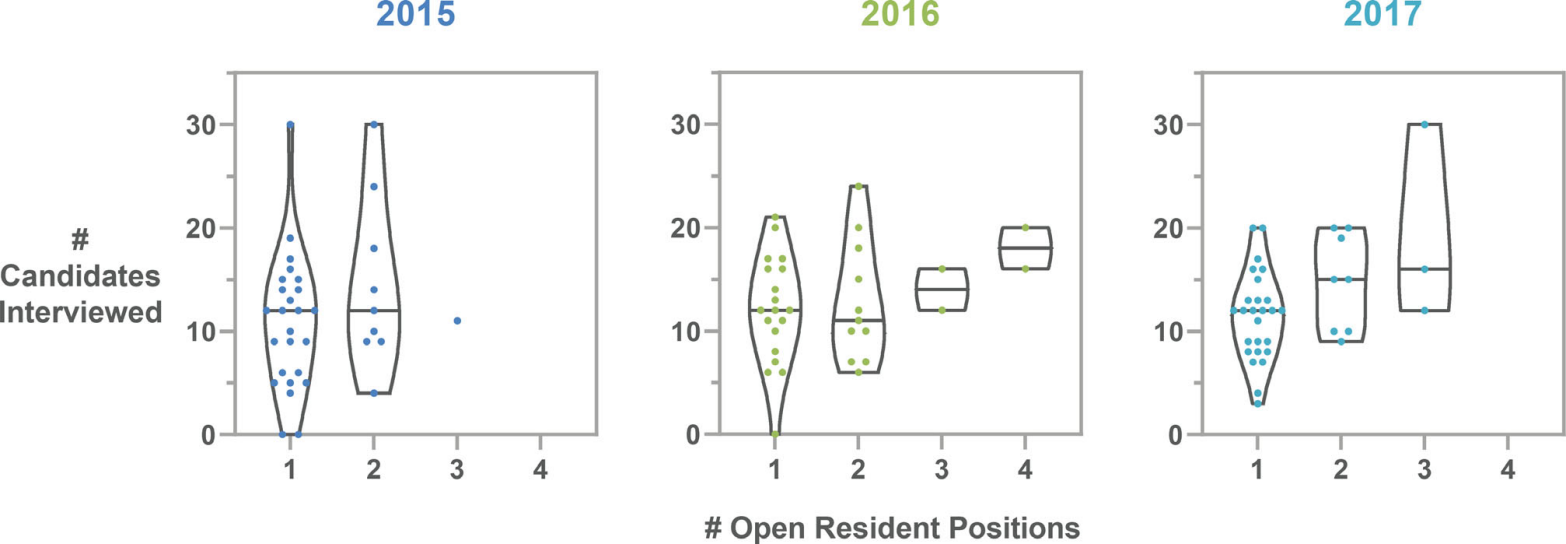
Number of candidates interviewed per number of residency positions offered for survey years 2015–2017 as reported by residency program directors. The horizontal line in each violin plot indicates the median number of candidates interviewed for each number of open positions. Each dot is an individual response.

PDs were asked how many residency positions they offered, and responses ranged from one to twelve, with most programs offering one residency position in the match (69%, 54%, 68% in 2015, 2016, and 2017, respectively). It is acknowledged that we are not aware of any program accepting twelve new applicants in a single year, and that the answer likely instead represents the total number of residents in the program. PDs were then asked how many of those positions were filled in the match that year; responses ranged from one to four. For those institutions who filled all of their positions in the match, PDs were asked how many applicants were interviewed on‐site. The range of responses is shown in Fig. 16, with average and median interviewees shown per number of residency positions offered. The average number of interviewees per position ranges from 10.8 to 19 for one to three open positions. In the 2017 survey, PDs were asked if they conducted remote (telephone or teleconference) interviews. Fifty percent of programs indicated that they did, with 85% using the process for screening interviews and 15% utilizing remote interviews as an option for final interviews. While on‐site interviews are clearly preferred, it is anticipated that many interviews will be performed remotely in the 2020–2021 match cycle due to the COVID‐19 pandemic.

Travel to participate in interviews can be costly for participants, as shown in Fig. 6. PDs were asked if their program provides financial assistance to candidates interviewing on‐site. Most programs provide meals during the interview (73%), while 20% provide hotel/housing, and 2% offer funds to offset travel costs. In addition, some programs provide transportation from hotel to the airport or interview site. Experiences in 2021 with virtual interviewing platforms may make this option more acceptable in the future and therefore more affordable for candidates.

Satisfaction with the match experience was previously reported,^7^ to which we add 2017 survey data. Ninety‐eight percent (2015), 92% (2016), and 85% (2017) of PDs report that they were very satisfied or satisfied with the match experience. Seventy‐five percent (2015), 75% (2016), and 60% (2017) report that “it is a reasonable process that needs no changes.” In the 2017 survey, PDs were additionally asked if they plan to continue to participate in the match; 92% responded “yes” and 8% responded “unsure.”

### 3.8 Current status of residencies in medical physics

**Fig. 17:**
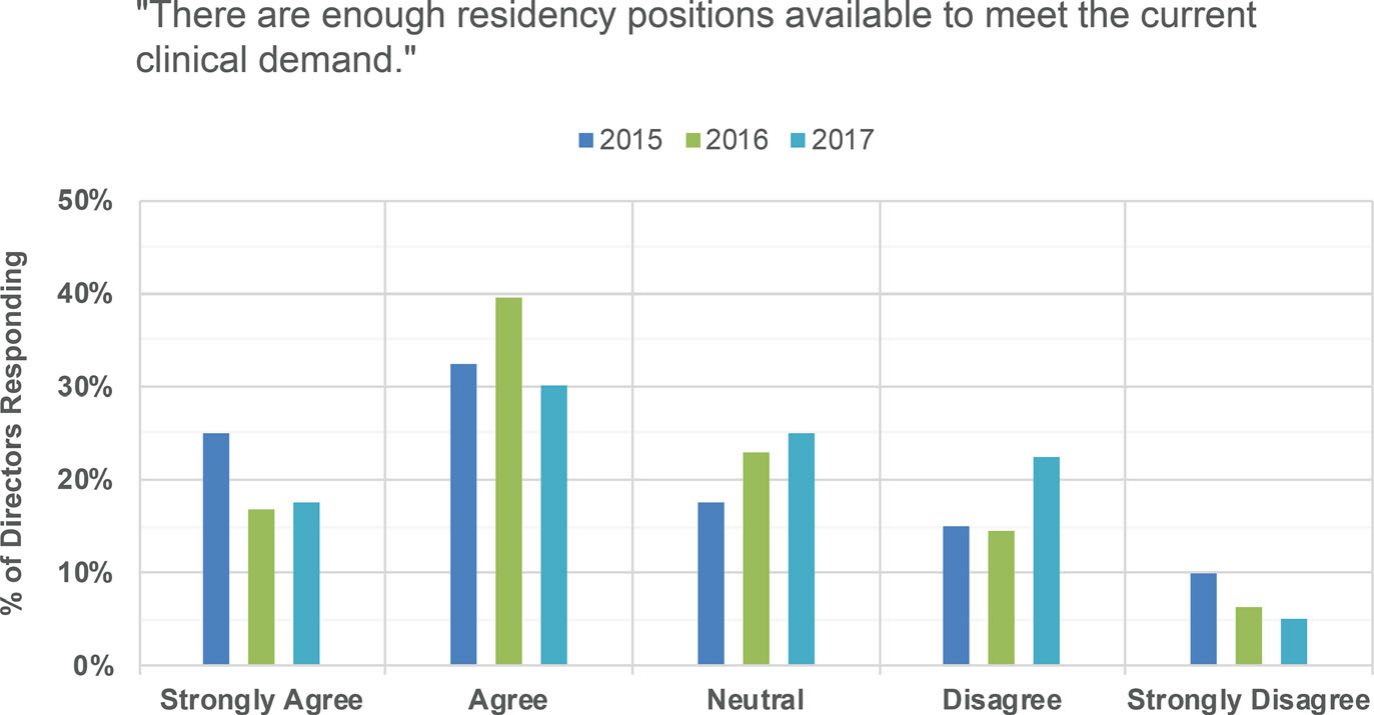
Program director responses to the statement “There are enough residency positions in available to meet the current clinical demand” for 2015, 2016, and 2017 surveys.

**Fig. 18:**
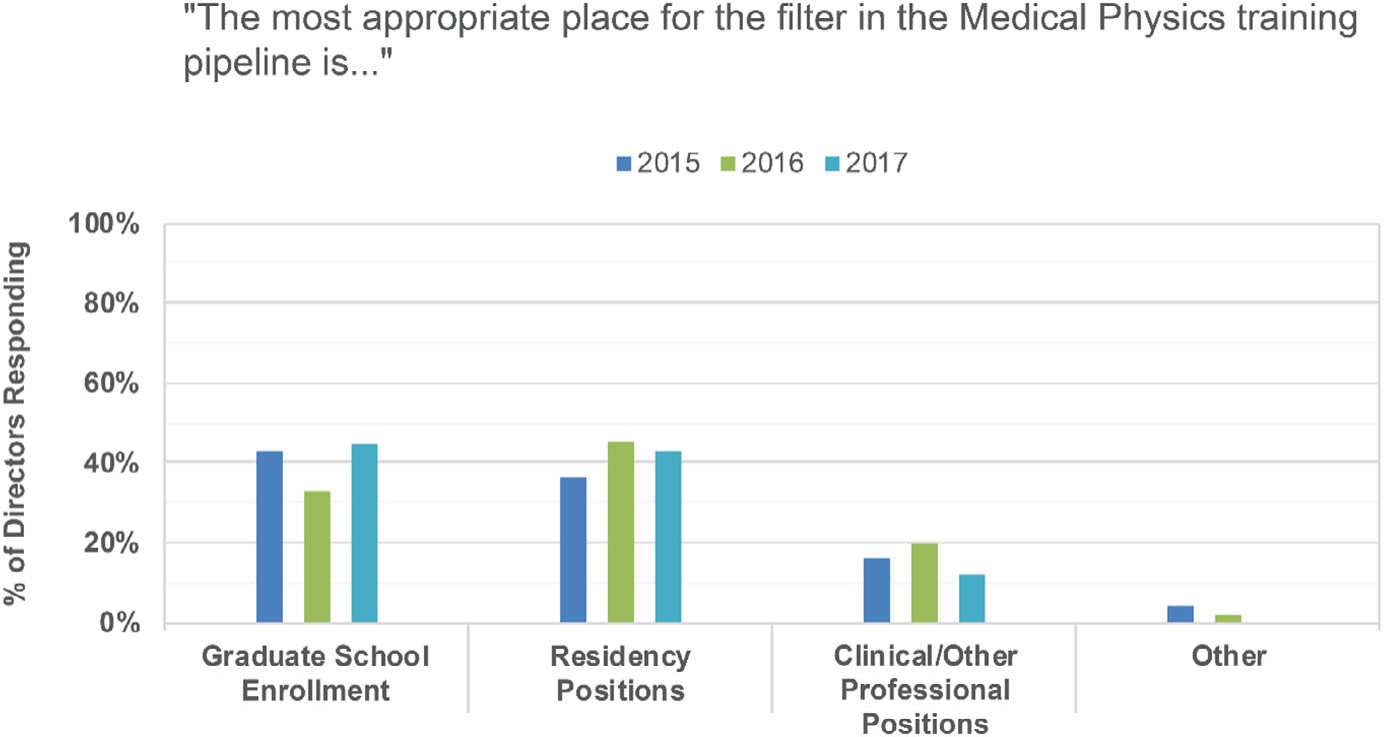
Program director responses to the statement “The most appropriate place for the filter in the Medical Physics training pipeline is…” for 2015, 2016, and 2017 surveys.

PDs were asked if they agree with the statement that there are enough residency positions available to meet current clinical demand (see Fig. 17) and where in the medical physics training pipeline is most appropriate for a filter (see Fig. 18). While applicants more strongly preferred graduate school enrollment as the appropriate filter location (Fig. 11), PDs indicated that graduate school enrollment and control over the number of residency positions to be roughly equally appropriate filter points (Fig. 18). In other medical fields, the number of available positions in medical school is controlled, thereby serving as the first and perhaps primary filter in selecting individuals into the medical profession. In some medical subfields, with radiation oncology as a recent example, residency slots remain unfilled by their match.^10^ In medical physics, there is currently no national or external control over the number of slots in medical physics graduate departments, and there are substantially fewer medical physics residency slots available compared to the number of graduates annually. While nearly all medical school graduates can be expected to acquire a clinical residency position and continue on to medical practice, medical physicists with graduate training do not all need to complete residency training in order to have a career in medical physics. There are career options available in industry and government, as well as some academic and clinical medical physicist jobs that do not require board certification and therefore do not require completion of a residency.

In 2015, 58% of PDs indicated that they agreed or strongly agreed that there are enough residency positions available, with 56% and 48% similarly agreeing in 2016 and 2017, respectively. In 2015, 25% of PDs indicated that they disagreed or strongly disagreed with this same statement, with 21% and 28% similarly disagreeing in 2016 and 2017, respectively. Finally, while an average of 66% of applicants agreed or strongly agreed that a residency position was difficult to obtain (Fig. 8), only 40%, 33%, and 38% of PDs saw the current residency placement rate as a problem for our profession in the 2015, 2016, and 2017 surveys, respectively.

While the number of programs and positions filled in the match were both higher in the 2020 MPM than in the 2019 MPM, the departure of a small number of high‐profile programs from the match has raised concerns about the trajectory of match participation. While this topic will not be discussed here, it is worth noting that the PD satisfaction rate from survey respondents shows a consistently decreasing trend (data presented in Interviews, Rank Lists, and Preferences section above). Both metrics related to match experience, including over all match experience and that the process needs no changes, decreased by 13% and 15% over the three years that program directors were surveyed.

Given the significant potential effects on our profession, the relative supply of and demand for medical physics residency positions is the topic of important consideration within our education and training infrastructure.^11^ The number of graduates in medical physics, the number of applicants to the MPM, and the relative success of those applicants has been closely monitored, and program graduate data and match statistics are publicly available.^2^, ^12^ It is useful to compare the results from our study with these data sources.

The percentage of respondents in our survey who were accepted into a residency program is given in Table 6 and averages 62% over the four years of survey data provided. This is significantly higher than previously published estimates.^2^ However, it should be noted that our data represents a small (roughly 30%), and potentially atypical, cohort of applicants. As an example, averaged over all four years, 87% of respondents to this survey submitted a rank list. In comparison, the percentage of all applicants submitting a rank list provided by the MPM can be estimated as the number “participating” in the match divided by the number “registering” for the match. These results are 70% (2015), 63% (2016), 77% (2017), and 70% (2018). The average of these values (70%) is substantially lower than the 87% from the survey data presented here. Over all, applicant match rates continue to climb, and data from the MPM shows that the percentage of candidates registered for the match who were successful in matching was 27%, 32%, 37%, and 43% in 2015 through 2018.^2^

Interestingly, data from the CAMPEP graduate program directors survey suggest a much higher rate of placement into residency positions. Including MS, PhD, and certificate program graduates, the CAMPEP graduate program directors survey indicates that 145 graduates applied to residency positions and 111 graduates were accepted into a residency in 2018.^12^ These data are more difficult to interpret since the graduates applying for residency in a given year are not necessarily the same graduates as those accepted into a residency in that year, and cannot be compared directly to the match data since they also include non‐match positions. However, there is clearly a major difference in the perceived success rate of graduates.

In addition, while there were 272 and 273 applicants registering for the match in 2017 and 2018, the CAMPEP survey data yield 130 and 118 MS and PhD graduates applying for residency in 2017 and 2018, respectively. Even including those coming from certificate programs, these data only represent approximately half of the applicants who registered for the match. Our 2018 survey data includes 17 respondents (19%) who had applied in a previous match cycle. Even if the over all percentage of match applicants who have applied to a previous cycle is higher than this, it is unlikely that this would be nearly enough to make up the disparity between number of graduates applying for residency from the CAMPEP survey and the number applicants to the match.

It is important to note that the discussion of the “residency bottleneck” overshadows the potentially optimistic state of the future demand for medical physicists. The Mills et al. 2010 analysis of future trends of supply and demand of radiation oncology physicists predicted that by 2020 approximately 125 new radiation oncology physicists would be needed annually, which closely matches the number of residency slots available annually.^13^ However, a more recent 2019 evaluation of the supply and demand^14^ concludes that the limited number of residency slots is indeed leading to a surplus of graduates with no pathway to board eligibility and that additional residency slots are needed to ensure a healthy supply of medical physics residency graduates to support the future demand identified in the 2019 analysis. The models predict that 250 residents will be needed annually by 2030 in radiation oncology alone — currently less than 150 CAMPEP accredited therapy residents are graduating per year.^15^ Optimistically, this need could be met if we linearly extrapolate historic residency program growth, resulting in a predicted growth of approximately ten residency programs a year. However, recent growth is mainly attributed to additional imaging residency slots, while therapy residency growth has decreased in the past couple of years. It is imperative for the health of our profession, and is our professional and ethical obligation, to accurately understand future workforce needs and to adapt our education and training infrastructure accordingly.

### 3.9 Statistical significance

The survey data presented in the text and displayed in figures and tables in many cases represent small numbers such that no rigorous statistical significance can be claimed. Discussions of the data presented point out trends and differences in the data without the ability to estimate uncertainties. The authors have presented our interpretations and invite the reader to judge the data for themselves.

## 4 CONCLUSIONS

Now having completed its sixth cycle, the MedPhys Match has become a valuable and important part of the medical physics training infrastructure. We present here the first comprehensive published survey data on applicant and program director experiences during participation in the MPM. Quantitative results from the first four cycles of the match are presented, including data on application, interview, cost, success, and perception of the match and its role in our profession. Data presented here can assist future applicants in determining the number of applications to submit, the number of interviews to attend, the cost of these interviews, and the considerations most important to program directors in their ranking decisions. It can assist unmatched applicants in determining the most valuable course of action for a successful potential reapplication, namely, to gain more education or research experience before reapplying. It can also assist program directors in estimating the number of applicants to invite for interviews, understanding why candidates may decline an invitation, and what considerations are most important to applicants in their ranking decisions.

These data also provide a longitudinal evaluation of the perceptions of both applicants and PDs regarding the state of the MPM and the state of the education and training infrastructure in medical physics. Perceptions of PDs regarding the match are important in understanding how to make the MPM most successful and valuable to our profession. Understanding applicant perceptions is also particularly important since assurance of a viable, stable, and successfully navigable career pathway is an important consideration for assuring a continued supply of high quality candidates into our profession. Finally, we hope that concerns and suggestions identified by this survey can lead to better transparency and understanding of the match process and an improved experience for both applicants and programs.

A summary of takeaway points are as follows:

- Increasing percentage of female applicants participating in the match
- Increasing percentage of PhD applicants participating in the match
- Average number of applications submitted per applicant is approximately 20, range up to 100
- Increasing average number of interview invitations received per applicant currently < 10 per year, range up to 40
- Top two reasons to decline interview invitations is cost of travel and scheduling conflicts with other interviews
- Average of 87% of applicants who submitted a rank list indicated that program/institution reputation and program structure/organization were top two reasons for ranking a program highly
- Approximate minimum of five on‐site interviews needed to successfully match, yet < 5 interviews can result in a match and > 10 interviews can result in no match
- Majority of applicants spend at least $1000 on interviewing
- Top two re‐applicant activities between match cycles are medical physics research and additional medical physics education, with both of these activities increasing the chance of re‐applicants receiving interview invitations
- Sixty‐nine percent of PDs consider both PhD and MS candidates while 31% of PDs consider PhD applicants only
- Top two considerations for interview invite are clinical potential and content/quality of reference letters, while top two considerations for ranking decisions are impressions from interview and personality fit

## CONFLICT OF INTEREST

The authors have no relevant conflict of interest to disclose.
